# Quality of Service Provision in Fog Computing: Network-Aware Scheduling of Containers

**DOI:** 10.3390/s21123978

**Published:** 2021-06-09

**Authors:** Agustín C. Caminero, Rocío Muñoz-Mansilla

**Affiliations:** 1 Department of Communication and Control Systems, Computer Science Engineering Faculty, National Distance University of Spain (Dpto. de Sistemas de Comunicación y Control, ETSI Ingeniería Informática, Universidad Nacional de Educación a Distancia, UNED), E-28040 Madrid, Spain; 2 Department of Computer Science and Automatic Control, Computer Science Engineering Faculty, National Distance University of Spain (Dpto. de Informática y Automática, ETSI Ingeniería Informática, Universidad Nacional de Educación a Distancia, UNED), E-28040 Madrid, Spain; rmunoz@dia.uned.es

**Keywords:** scheduling, fog computing, network, quality of service, Kubernetes

## Abstract

State-of-the-art scenarios, such as Internet of Things (IoT) and Smart Cities, have recently arisen. They involve the processing of huge data sets under strict time requirements, rendering the use of cloud resources unfeasible. For this reason, Fog computing has been proposed as a solution; however, there remains a need for intelligent allocation decisions, in order to make it a fully usable solution in such contexts. In this paper, a network-aware scheduling algorithm is presented, which aims to select the fog node most suitable for the execution of an application within a given deadline. This decision is made taking the status of the network into account. This scheduling algorithm was implemented as an extension to the Kubernetes default scheduler, and compared with existing proposals in the literature. The comparison shows that our proposal is the only one that can execute all the submitted jobs within their deadlines (i.e., no job is rejected or executed exceeding its deadline) with certain configurations in some of the scenarios tested, thus obtaining an optimal solution in such scenarios.

## 1. Introduction

In recent years, distributed heterogeneous computing resources have arisen. Cloud computing can be considered as the application of business models existing in traditional utilities (e.g., water or power supply) to computing equipment, where users consume virtualized storage or compute resources offered by a provider, based on a pay-as-you-go model. The resource providers are large data centers with high computing, memory, and storage capacity. In addition, devices at the edge of the network can serve as part of Internet of Things (IoT) deployments. These can be sensors and devices that are part of Smart City infrastructure, lifestyle gadgets such as wearables, and smart appliances or smart phones. These devices were originally designed to sense and generate data streams, and have some spare capacity (computing, memory, and storage) which can be harnessed to execute applications [1].

**Figure 1:**
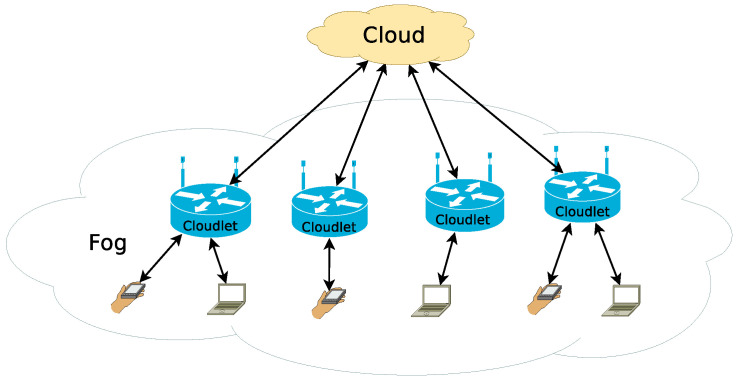
Multi-level edge/fog/cloud scenario [5].

The most recent term to be coined is fog computing, which refers to computing resources that are between the edge and cloud in the network hierarchy, with compute, storage, and memory capacities that fall between these layers [2]. These edge and fog resources provide the opportunity for low-latency processing of the generated data closer to its source, utilizing the wide-area network [3]. As a result, there is critical need to understand how this diverse ecosystem of edge, fog, and cloud resources can be effectively used for large-scale distributed applications [4]. This multi-level scenario can be seen in Figure 1 [5], where the term cloudlets refers to the access point devices, extended with computing and storage services to create the fog system.

In such a multi-level environment, the network plays an essential role, as it is the communication medium between all the actors in the system; as such, its performance affects the whole system. Thus, the network should be taken into account when making all kinds of decisions, in order to provide Quality of Service (QoS), with one of the main decisions being scheduling. The term “scheduling” refers to the process of allocating computing resources to an application and mapping constituent components of that application onto those resources, in order to meet certain QoS and resource conservation goals [6].

Virtualization technologies allow for the packaging and execution of applications in isolated environments, independent of the underlying hardware resources. The most traditional virtualization technology is hardware virtualization, which creates virtual machines (VMs)—abstractions of hardware, on top of which an operating system runs. They require extensive hardware resources to run, as VMs run full operating systems. A more recent method of virtualization is operating system-level virtualization, which creates containers [7]. They use the host operating system and, thus, do not require one for each container (as is the case for VMs), such that the hardware requirements to run containers are lower than for VMs. Thanks to this, containers are suitable to run on low-cost computers, such as the Raspberry Pi.

In order to deploy containers, a cluster of computers can be used. In this case, there is a need to manage the cluster, which is done by a container orchestrator; one of the best-known orchestrators is Kubernetes [8]. Among the tasks the orchestrator has to perform, one of the most important is scheduling the containers into the nodes of the cluster.

In the context of fog computing and IoT, Ficco et al. [9] have presented a number of open challenges, one of them being the development of new scheduling algorithms for the efficient use of fog resources. This is the challenge that our work tackles. Our work takes into account the deadlines of the tasks and the status of the interconnection network, in order to make the most appropriate decision about the fog node used to run each task. Other proposals from the literature that share the same objective are reviewed in next section.

The contributions of our work are as follows: (1) The development of an extension to the Kubernetes default scheduler, which allows it to use the network status to make decisions; (2) the development of a network-aware scheduling algorithm for fog environments (called the IPerf Algorithm, IPA), which calculates predictions for the execution time of applications and rejects those applications for which the system decides the deadline cannot be met; and (3) a performance evaluation of our algorithm is carried out, comparing it with other proposals from the literature.

The remainder of the paper is structured as follows. Section 2 presents the related work. Section 3 details the work done to extend the Kubernetes default scheduler. Section 4 provides our proposal for QoS provision in fog scenarios. Section 6 concludes the paper and suggests future work.

## 2. Related Work

The provision of Quality of Service (QoS) in fog computing environments has been reviewed by Kashani et al. [10], who identified several key categories; namely, service/resource management, communication management, and application management. Our work considers the first of these. Several proposals lying in this category are reviewed in this section. Furthermore, QoS provision in parallel-distributed computing systems has received a lot of attention from the research community over the years and, so, some significant works in this line are also reviewed in this section.

Skarlat et al. [11] presented a placement technique for IoT services on fog resources, taking into account their QoS requirements (including deadlines). Their proposal takes into account both cloud and fog resources. According to the authors, it prevents QoS violations and leads to a significantly lower cost of execution, if compared to a purely cloud-based approach. This approach takes into account the status of the network—more specifically, the network link delays. Among its weaknesses, this proposal does not reject applications when the deadline cannot be met (in contrast with the approach proposed in our work). Instead, when the deadline is close, the application is given priority over other applications with wider deadlines.

Aazam et al. [12] provided a resource management framework for IoT. Their model covers the issues of resource prediction, customer type-based resource estimation and reservation, advance reservation, and pricing for new and existing IoT customers, on the basis of their characteristics. Murtaza et al. [13] presented a task scheduling proposal that chooses the fog node which is most suitable for Internet of Everything (IoE) requests, in order to improve the response time, processing time, and energy consumption in fog environments. Neither of these proposals took deadlines of applications into account when making scheduling decisions, and were evaluated by means of simulations using CloudSim [14] and iFogSim [15].

Maiti et al. [16] considered the challenge of allocating service requests to Virtual Machines (VMs) running on different fog nodes under QoS restrictions. The use of VMs, rather than containers, imposes a heavy load on the fog resources; for this reason, we consider containers in our work. As in the works reviewed previously, this work did not take into account the deadlines of applications, and was also evaluated through simulations using MATLAB. Besides, this work did not take into account the status of the interconnection network.

Intharawijitr et al. [17] proposed three different strategies for optimum resource utilization. First, a random methodology was used to select the fog nodes to execute tasks upon arrival. Second, the focus was on the lowest latency fog devices. Finally, the fog resources with the maximum available capacity were primarily considered. Although the second strategy took transmission delays into account, it dids not consider deadlines. The three proposed policies were then evaluated, using a mathematical model.

Zeng et al. [18] introduced a joint optimization task scheduling and image placement algorithm, which was designed to minimize the overall completion task time for better user experience. The first sub-problem investigated was to balance the workload in both client devices and computational servers. The second sub-problem investigated was the placement of the task image on storage servers, while the final sub-problem was to balance the input–output interrupt requests among the storage servers. Although the transmission delays were considered, in order to calculate a completion time for tasks, no deadline was considered. This proposal was also evaluated through simulations.

The proposals that share more similarities with ours are the algorithm presented in [19] and the Network Aware Scheduler (NAS) algorithm, presented in [20] and extended in [21]. Regarding [19], the authors presented a scheduling algorithm for Kubernetes, which relies on the characterization of the incoming applications to be deployed on the cluster, based on their use of resources. Their intention was to avoid applications with high resource usage (CPU or disk) being deployed on the same node. They classified applications based on high or low use of resources, and considered two resources: CPU and disk. Classification of the applications is performed by the client or developer of the application to be deployed. Regarding [21], the authors presented a network-aware scheduling algorithm for fog computing. For this, nodes in the Kubernetes cluster are labeled with their Round Trip Time (RTT) and bandwidth. For the incoming pods to be scheduled, they include a minimum bandwidth label, which is used to filter out nodes which do not have the necessary bandwidth available. After that, the node with the lowest RTT is chosen to deploy the pod. These two proposals were tested using a real Kubernetes clusters.

As mentioned above, these proposals share some drawbacks. First, some of them do not take the network status into account when making scheduling decisions. Second, none of these proposals decided about whether an application can be executed within its deadline or not; as such, all the applications submitted to the system are accepted and executed. Third, some of the above referenced proposals were evaluated by means of simulation or mathematical evaluation, rather than through the use of a real implementation.

The work presented here tackles these drawbacks, as follows: Our system uses information about the status of the network—namely, the bandwidth and round trip time (RTT)—to make scheduling decisions. Our system predicts whether a application can be executed within its deadline and rejects applications when the system decides their deadlines cannot be met. Finally, a evaluation conducted using the extending Kubernetes scheduler is presented.

## 3. Extending Kubernetes Default Scheduler

**Figure 2:**
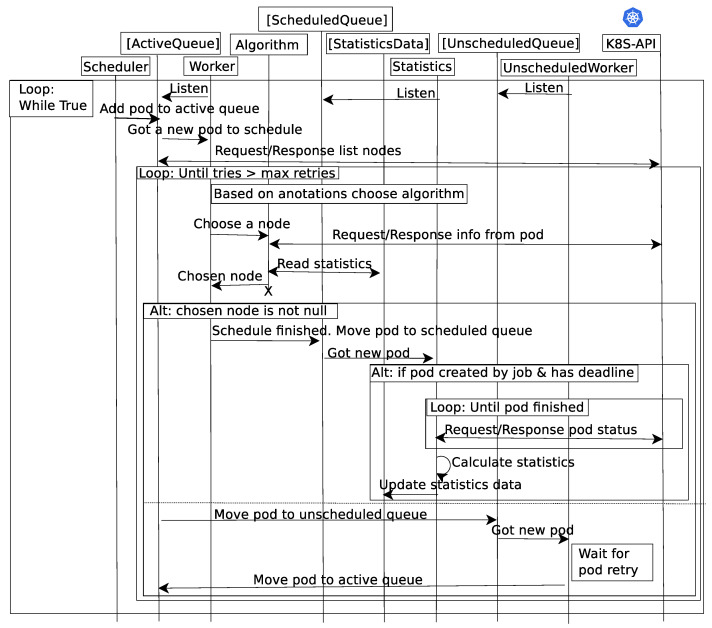
Sequence diagram.

In order to allow Kubernetes to deploy scheduling algorithms using network status information, a new scheduler module was developed in the Python language, whose main components and interactions are depicted in Figure 2. Different threads are used to separate different functionalities. For communication between threads, queues are used.

In the image, the Scheduler thread is called when a job is submitted to Kubernetes, and it includes this new pod in the ActiveQueue, which the Worker thread listens to. This thread retrieves the list of available nodes from the K8S-API and decides which scheduling algorithm should be used to handle this pod. For the selected algorithm, the thread Algorithm retrieves information of the pod from the K8S-API and statistics for the previous executions of this pod. Using this information, the Algorithm thread chooses a node to execute this pod, and returns it to the Worker node. The following section details the Algorithm thread, which is one of our main developments.

If a node has been selected to run the pod, it is included into the ScheduledQueue, and the Statistics thread polls the K8S-API for the status of the pod. Once the pod completes its execution, statistics are calculated, including the completion time of the pod. This statistic is used by the Algorithm thread for future scheduling decisions.

If no node is selected, the pod is moved to the UnscheduledQueue, where it waits for a new scheduling decision. There is a number of retries, until the pod is eventually rejected or accepted for execution.

In order to allow the scheduling algorithms to use the network status information, iperf [22] is used to calculate the network status. An iperf server was deployed as a docker container running in each worker node, and a central component was developed that tests every worker at a configurable frequency (which, by default, is set to 30 s). This component runs on the master node of the cluster, and adds the network information as metadata of the nodes; as such, it is available through the API of Kubernetes.

Furthermore, in order to allow the ability to schedule each pod with a different scheduler, the information of the chosen scheduler algorithm for a pod is included in the manifest.

In order to implement this extension, the architecture of a Kubernetes cluster has been modified. Originally, a Kubernetes cluster has two parts [23], namely the control plane and the nodes, all of them communicated through a network connection.

The control plane has the following components:

- API: the front end for the Kubernetes control plane.
- etcd: highly-available key value store for all cluster data.
- sched: it matches pods to nodes.
- c-m or controller manager: it runs controller processes, such as jobs or node controllers.
- c-c-m or cloud controller manager: if the cluster is deployed in a cloud provider, it links the cluster into the cloud provider’s API.

The nodes have the following components:

- k-proxy or kube proxy: it maintains network rules on nodes.
- kubelet: it ensures that the containers are running according their specifications.

**Figure 3:**
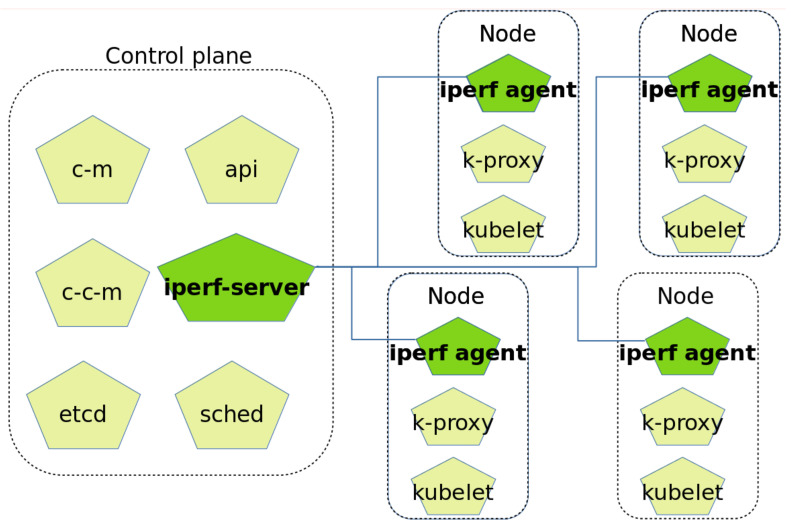
Architecture of a Kubernetes cluster with the extensions.

Two new components have been added to this architecture in order to implement our new functionality. These are one iperf-server per cluster and one iperf-agent per node. The iperf server monitors network status of the nodes and then publishes its information in the API. This architecture is presented in Figure 3, where the new components appear in green.

### Algorithm Class

This class takes the nodes of the cluster and the pod to schedule, creates a connection to the API of Kubernetes, and initiates the logging. This class is shown in Listing 1.

To develop a new scheduling algorithm, the evaluate method must be overridden, in order to chose the most appropriate node to run a pod. The name of the class is used in the manifest of the pods to be scheduled, after the scheduler/algorithm: annotation.


Listing 1: class Algorithm().

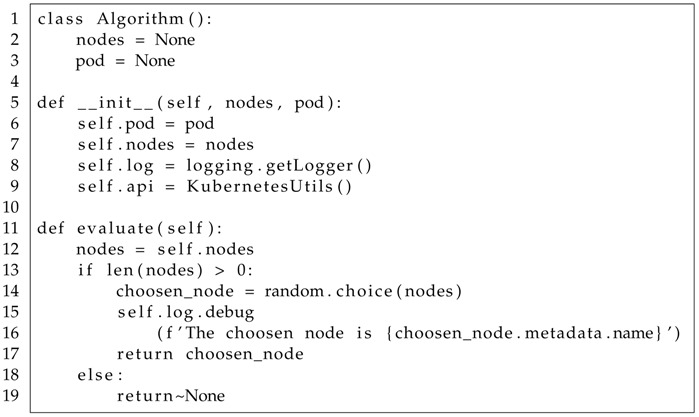

## 4. Proposal for QoS Provision in Fog Computing: IPerf Algorithm (IPA)

Using the extension to the Kubernetes scheduler presented in the previous section, a proposal for fog computing scheduling is presented, which takes into account the status of the network to map pods to the nodes of the cluster that are more suitable to run them within their deadline. This algorithm is called the IPerf Algorithm (IPA), which is presented as pseudocode in Algorithm 1.

This algorithm is aimed at applications that have strong network consumption and utilize deadlines. It has two phases: filter and rank. In the filter phase, it is checked whether the pod to be scheduled is part of a job [24] and has a deadline. This is shown in line 9 in Algorithm 1. In Kubernetes, a job creates one or more Pods and ensures that a specified number of them successfully terminate. As pods successfully complete, the Job tracks the successful completions. When a specified number of successful completions is reached, the job is complete. Jobs may define the directive activeDeadlineSeconds, which applies to the duration of the job, no matter how many pods are created. Once a Job reaches activeDeadlineSeconds, all of its running Pods are terminated. If this test is true, then the available nodes in the Kubernetes cluster are checked, in order to decide whether they can run the pod within its deadline or not, by calling the function filter_nodes_statistics (line 10). This function is explained in Algorithm 2. As a result of this first phase, the only remaining nodes of the system are ones suitable to running the pod within its deadline, which are used as inputs for the second phase.

In the rank phase, a quality is calculated for each suitable node (line 14), which takes into account the use of the resources by the existing pods running in the node. This function is explained in Algorithm 3. Then, the node with the highest quality is chosen to deploy this pod (line 21).
**Algorithm 1** IPerf Algorithm (IPA).1:Let nodeList: list of active nodes in the system2:Let pod: a pod to be scheduled3:Let suitableNodes: list of nodes that could run the pod within its deadline4:Let chosenNode: the node that the scheduler chooses for this pod5:Let bestQuality: the highest quality of the already tested nodes6:Let quality: the quality of a node7:bestQuality = 0.08:/* Filter phase */9:**if** (pod is part of a Job) and (pod has activeDeadlineSeconds) **then**10: suitableNodes = filter_nodes_statistics(nodeList, pod)11:**end if**12:/* Rank phase */13:**for all** node in suitableNodes **do**14: quality = calculate_quality(node) 15: **if** quality > bestQuality **then**16:  bestQuality = quality17:  chosenNode = node18: **end if**19:**end for**20:**if** chosenNode not null **then**21: assign(pod, chosenNode) 22:**end if**

The function to filter nodes is presented in Algorithm 2. It receives the list of nodes available in the system and pod to be scheduled as input. For each node, it calculates an estimate for the completion time of the pod using the node, and removes it if this estimate is not within the deadline of the pod. This estimation is calculated as presented in Algorithm 4, where the previous executions of the pod in the node are considered.
**Algorithm 2** filter_nodes_statistics(nodeList, pod) function.1:Let nodeList: List of nodes available in the system2:Let $deadline_{pod}$: The deadline of pod3:**for all** node in nodeList **do**4: **if** CompletionTimeEstimation(pod, node) $>deadline_{pod}$ **then**5:  remove node from nodeList6: **end if**7:**end for**8:return nodeList

**Algorithm 3** calculate_quality(node) function.
1:Let weight_rtt: weight of the rtt in the schedule decision2:Let weight_bandwidth: weight of the bandwidth in the schedule decision3:Let weight_memory: weight of the % memory in the schedule decision4:Let weight_cpu: weight of the % cpu in the schedule decision5:Let weight_current_pods_type: weight of the number of pods of the same type running in a node in the schedule decision6:Let quality: the quality of a node7:rtt = normalize_rtt(node) * weight_rtt8:bandwidth = normalize_bandwidth(node) * weight_bandwidth9:cpu = normalize_cpu(node) * weight_cpu10:memory = normalize_memory(node) * weight_memory11:current_pods_type = normalize_current_pods_type(node) * weight_current_pods_type12:quality = rtt + bandwidth + cpu + memory + current_pods_type13:return quality


**Algorithm 4** CompletionTimeEstimation(pod, node) function.
1:Let node: a node in the system2:Let pod: pod to be scheduled3:Let *n*: the number of samples of completion time for the pod in the node4:Let $CompletionTime{\left(pod,node\right)}_{j}$= the $j_{th}$ completion time for the pod pod in the node node5:
$CompletionTimeEstimation=\frac{\sum_{j=1}^{n}CompletionTime{\left(pod,node\right)}_{j}}{n}$
6:return CompletionTimeEstimation


Algorithm 3 details how the quality of nodes is calculated. It is done by taking into account several parameters for each node; namely, the percentage of CPU and memory occupation in the node, the number of pods already deployed on it, and the bandwidth and round trip time (RTT) of the node. As can be seen, the network characteristics are considered for this decision. Each parameter has a weight, which can be configured. For each parameter, a normalization function is called (presented in Listing 2). Each normalized parameter is multiplied by its associated weight (lines 7 to 11). Then, all the parameters are added, in order to calculate the quality of the node (line 12).

The normalization functions used in Algorithm 3 are presented in Listing 2. These functions consider each resource (RTT, bandwidth, CPU, memory, running pods), and they aim at giving a high value when the utilization of the resource is lower. Therefore, for the CPU and memory (which are measured as percentages of utilization), they must be reversed, in order to show the availability of the resource—this is done by taking 1 minus the utilization of that resource (see lines 23 and 28).

For the RTT and bandwidth, these parameters are normalized by operating them along with the lowest and highest values for each parameter (lines 8 and 18). The current number of pods already running in the node is also normalized, using the configured maximum number of pods in a node (line 33).


Listing 2: Normalization functions.

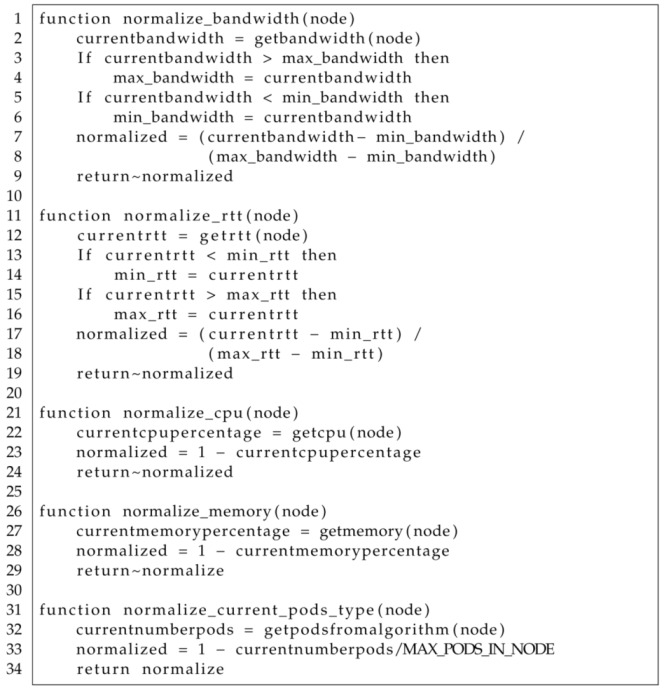

### 4.1. Complexity Analysis

The time complexity of the proposed IPA algorithm was analyzed, and the results are as follows. The normalization functions presented in Listing 2 and the calculate_quality function, presented in Algorithm 3, have constant complexity. The CompletionTimeEstimation function, presented in Algorithm 4, has O(n) complexity, which depends on the number of existing samples. This function could also be implemented with a constant complexity if, instead of storing all the samples for the completion time of a pod in a node, only the addition of all the samples and the counter of samples were stored.

The filter_nodes_statistics function, presented in Algorithm 2, also has O(n) complexity, which depends on the number of nodes in the system. However, this function can be executed in parallel, in the case where the number of nodes in the system grows, by devoting more than one server to host the scheduler and splitting the information on the nodes between these servers, such that each one performs this function on a subset of the nodes.

## 5. Performance Evaluation

In this section, a performance evaluation of the IPA algorithm is presented, through a comparison with several other proposals from the literature, which are detailed in Section 2. Authors considered that the best comparison would be against the most up-to-date proposals from literature, specifically those which are also network-aware (similarly to IPA), rather than against well-known algorithms such as Shortest Job First (SJF) which are not network-aware.

Regarding the proposals from the literature, we compared the IPA algorithm with the algorithm presented in [19] and the NAS algorithm [21]. Both are reviewed in Section 2. The algorithm of [19] was modified to implement network awareness (labeled in the figures as CA-Net; Contention-Aware Network), which was done by using a disk tag for the use of the network; all the jobs submitted share a high load for this resource. So, all the proposals tested implemented network awareness. The manifest for the IPA algorithm tested is presented in Listing 3.

The evaluation has two main parts, first the complexity of the algorithms is compared, then a performance evaluation is presented. Regarding the first part, Section 4.1 presents the complexity of IPA. With regard to NAS algorithm, it is a recursive approach as presented in [21]. When a pod enters the system, NAS first checks all the nodes in the system in order to find the node with the lowest RTT. This part of the algorithm has O(n) complexity depending on the number of nodes in the system. After that, it finds the bandwidth of that node and checks if both RTT and bandwidth are within the requirements of the pod. This part also has O(n) complexity depending on the number of nodes in the system. If the node with the lowest RTT does not have enough bandwidth for the new pod, then the whole procedure is repeated recursively eliminating this node from the list of available nodes. Thus, the complexity of NAS can be seen as $nO^{2}\left(n\right)$ depending on the number of nodes in the system. Regarding CA algorithm as presented in [19], its complexity is O(n) depending on the number of nodes in the system. Considering this information, it can be seen that CA is the least complex algorithm, followed by IPA and finally NAS.

Now we move on to the performance evaluation. This evaluation was performed using a testbed based on Kind [25], which was used to create a cluster made of one master and four worker nodes, all of them running on the same physical server. Figure 3 represents our testbed graphically. The application used in the tests was a speed test [26], which was deployed in a docker image developed by the authors [26]. This application was chosen as it requires high bandwidth consumption. This application performs a test of network speed, where the number of times that it is repeated is a configurable parameter; in our case, this was set to 2.

The tests conducted consisted of 200 jobs, submitted with three seconds between each one and with three different deadlines (60, 100, and 120 s). These deadlines were chosen such that the jobs were difficult to meet and, so, the chosen scheduling algorithm could make a difference. The network-monitoring interval was set to 30 s and the minimum number of executed jobs to calculate predictions was set to 10.

IPA was evaluated using several different configurations: IPA-Original (labeled IPA-O in figures), IPA-Network (labeled IPA-N), IPA-Balanced (labeled IPA-B), and IPA-Equal (labeled IPA-E). These are shown in Table 1. For these configurations, the weights assigned to each parameter varied, where network bandwidth and existing pods running on the node always had a significant weight.


Listing 3: IPA manifest.

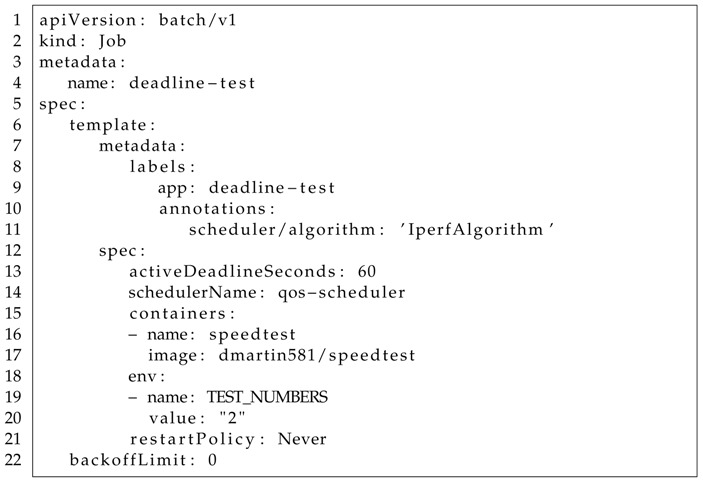

**Figure 4:**
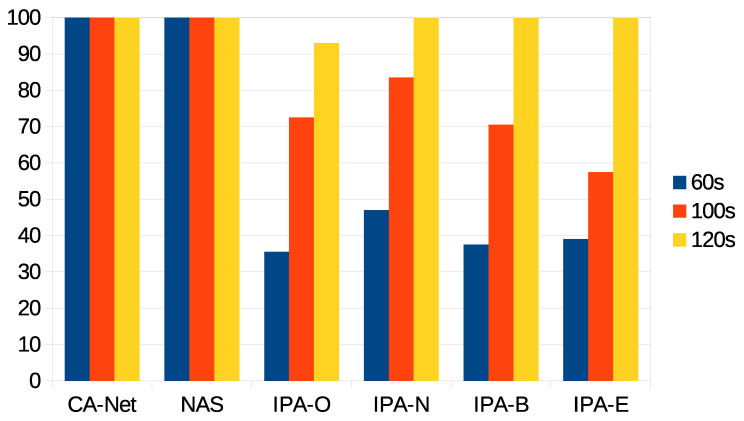
Percentage of accepted jobs for each deadline.

The first statistic to be presented is the percentage of jobs accepted for execution for each algorithm and each deadline. For the different configurations of IPA, these are jobs whose estimated completion time was within the deadline. This statistic is shown in Figure 4, from which is can be seen that the CA-Net and NAS algorithms scheduled all the jobs that were submitted to the system, as they do not perform any kind of admission control. In constrast, the different configurations of IPA rejected some pods, when the prediction of the completion time for the application was beyond the deadline. As can be seen, the wider the deadline was, the more pods were accepted for execution. This was because, at the beginning of the experiments, there were no data on the execution time of jobs and, so, no predictions could be calculated. In this case, the jobs were accepted and assigned to random nodes. Only when a minimum number of jobs had successfully finished their executions, could their completion times be used to calculate predictions for the completion times of future jobs. Therefore, CA-Net and NAS were between 52% and 64% better than any configuration of IPA for the 60 s deadline, between 18% and 42% better for the 100 s deadline, and 7% better than IPA-O for the 120 s deadline.

It can also be seen that different configurations of IPA showed slightly different results. IPA-N accepted more jobs under the 60 and 100 s deadlines (between 8% and 12% more than the other configurations of IPA for the 60 s deadline, and between 11% and 21% for the 100 s deadline). IPA-E accepted fewer jobs for the 100 s deadline. Furthermore, IPA-O was the only configuration that rejected some jobs with the 120 s deadline (7% of jobs). These differences were related to the monitoring interval of the network, which was set to 30 s. Therefore, the use of the network caused by the execution of jobs is only considered for future scheduling decisions after the network has been monitored, which happens at most 30 s after the job execution has started. Thus, scheduling decisions made in the meantime do not reflect the current status of the network. A decrease in the monitoring interval may be considered, but it should be noted that monitoring the network involves sending synthetic information through it, which may affect real transmissions. So, a trade-off between the monitoring interval and accuracy in the status of the network must be reached.

**Figure 5:**
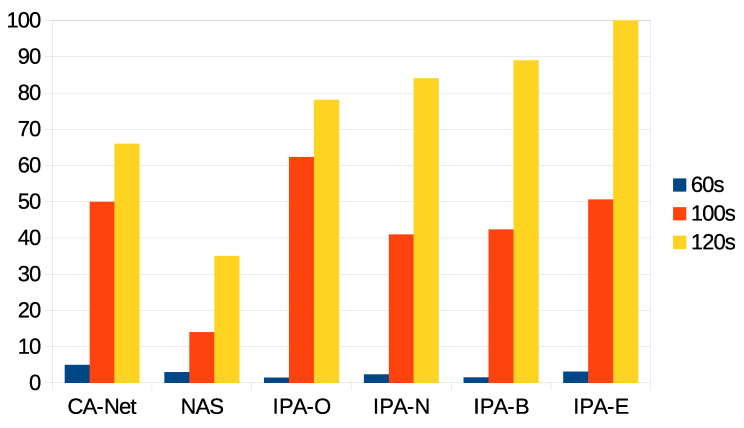
Percentage of successfully executed jobs for each deadline, out of the submitted jobs.

The next statistic presented is the percentage of jobs that were successfully executed, out of the total amount of jobs submitted to the system, as presented in Figure 5. It can be seen that, although CA-Net and NAS accepted all the jobs, a low percentage of them met the deadline (at most 65% for the 120 s deadline). This was expected, as they did not make decisions based on the deadlines of jobs. Furthermore, the longer the deadline is, the more jobs are successfully executed. The algorithm with the lower values was NAS, which was between 27% and 48% worse than any configuration of IPA for the 100 s deadline, and between 50% and 65% for the 120 s deadline. One reason for this behavior is that, although it uses the status of the network to make scheduling decisions, it does not calculate predictions for the completion times of jobs. In contrast, CA-Net tries to distribute the workload among all the nodes, regardless of their actual use of resources. Recall that, in CA-Net, jobs require labeling, which is performed by the client application; in our experiments, all of the jobs were labeled as requiring high network use.

**Figure 6:**
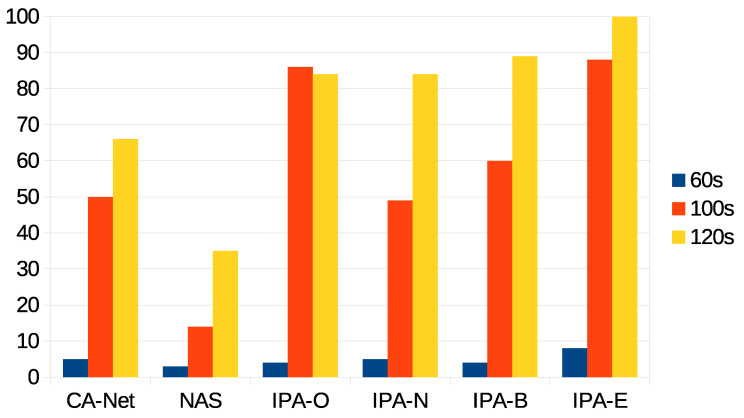
Percentage of successfully executed jobs for each deadline, out of the accepted jobs.

Next, we present the percentage of accepted jobs that were successfully executed for each deadline, as shown in Figure 6. This is the main statistic illustrating the success of the IPA proposal when providing QoS to applications deployed in the cluster, as this statistic indicates the correctness of a decision to accept a job.

It can be seen that, for the tightest deadline (60 s), few jobs could be successfully executed for all the algorithms (the best being IPA-E, with less than 10% of successful jobs), leading to poor QoS. For all the IPA configurations, too tight deadlines affected them heavily, as it cannot calculate predictions on future pods—IPA can only calculate predictions for future jobs when a minimum number of previous jobs are successfully executed.

For the 100 s deadline, IPA-O and IPA-E obtained the best results: between 26% and 39% better than the other configurations of IPA, more than 35% better than CA-Net, and more than 70% better than NAS. IPA-N presented similar results as CA-Net, while the worst results came from NAS. The results from CA-Net suggested that the mere labeling of applications as high or low consumers of a resource is not enough to provide QoS, as deadlines of applications are not considered at all. As IPA-N and NAS gave the worst results, this suggests that using the network alone to perform scheduling does not provide QoS to applications; even though, in this case, real measurements of the status of the network were used, instead of relying on the labeling of applications.

For the 120 s deadline, all the configurations of IPA outperformed the other proposals. There were slight differences between IPA-O, IPA-N, and IPA-B, which were around 15% better than CA-Net and almost 50% better than NAS. The best results were obtained using IPA-E, which showed 100% of succeeded jobs. Recall that IPA-E was able to schedule all the jobs and, so, all the jobs submitted to the system were successfully executed within their deadline, for the 120 s deadline, with this configuration. This was the only configuration that could meet the deadline of all the jobs submitted to the system, and it can be concluded that IPA-E provided an optimal solution in this scenario, being the only proposal that could achieve this.

For IPA-N, almost 15% of jobs were not executed within their deadlines. This highlights the fact that the network alone is not a good candidate to make scheduling decisions.

**Figure 7:**
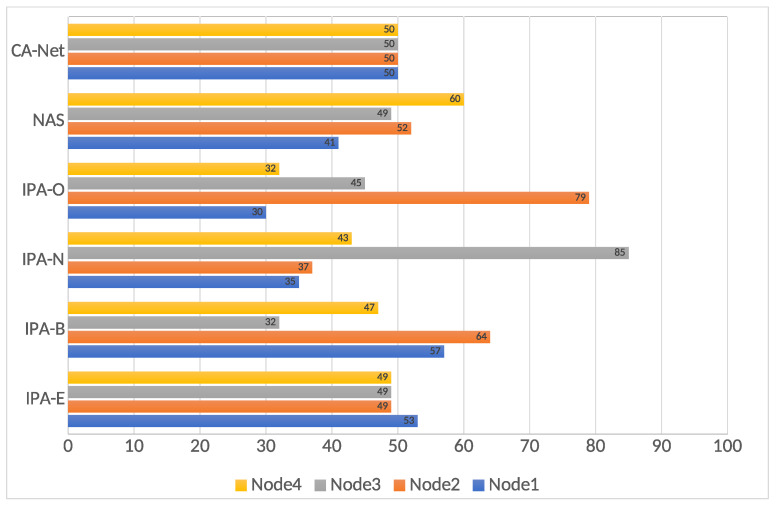
Jobs executed in each node, for the 120 s deadline.

In order to better understand these results, a final statistic is presented: the allocation of jobs to nodes. This is presented in Figure 7. This is only presented for the 120 s deadline, as the most noticeable differences were found for it. It can be seen that, for IPA-E, all the nodes executed a similar number of jobs, in the same way, as CA-Net. However, as presented above, IPA-E presented better results, regarding successfully executed jobs, than CA-Net (more than a 30% increase). These better results originate from the way that IPA-E tries to balance jobs between nodes, as it calculates a quality involving the network and the jobs already running in a node, while CA-Net tries not to submit two jobs of the same type to the same node. Considering that all the jobs were of the same type (all of them involving heavy use of the network), CA-Net chose nodes almost at random all the time.

## 6. Conclusions and Future Work

Fog computing has arisen in recent years, providing a more suitable solution than cloud computing when there are time restrictions for the processing of large data sets, such as those found in IoT applications. There are a number of open challenges in such fog and IoT ecosystems, one of them being the development of new scheduling algorithms for the efficient use of fog resources. In this context, this paper presented a scheduling algorithm for fog computing which has the following main features: First, it was designed to provide Quality of Service (QoS) to applications; which, in our case, involves executing those applications within a deadline. Second, it makes scheduling decisions while taking into account the status of the network. Third, it calculates estimates for the completion time of jobs. The proposed scheduler, named the IPerf Algorithm (IPA), was implemented as an extension to the Kubernetes default scheduler. Several variations of IPA were tested, in which the weights of different parameters varied, and compared with different proposals from the literature. For the highest deadline, when IPA makes decisions considering the network and the jobs already running as having equal importance (i.e., IPA-E), it can successfully execute all the jobs submitted to the system within their deadlines, providing at least a 30% increase over the other proposals from the literature.

This work provides a number of contributions. First, it was shown that the scheduling algorithm plays an essential role in QoS provision. Second, we demonstrated that the network status information should be taken into account when performing scheduling but always combined with other parameters. Third, estimates of the completion time of jobs should be calculated, in order to decide whether a job can be executed within its deadline. Fourth, we proposed a scheduling algorithm called IPA, and found that one of its configurations—called IPA-E—can provide an optimal solution for one of the tested scenarios; making it the only scheduling algorithm that could achieve an optimal solution in any of the tested scenarios.

As for future work, improvements to IPA can be implemented, which may involve, among others, calculating predictions in a more efficient way, and include concepts from autonomic computing in order to improve the accuracy of the predictions, in the same way as [27]. Furthermore, scheduling in advance is another interesting concept, such that jobs are submitted to the system and scheduled some time before the start of their execution, in the same way as [28]. Also, the use of other network metrics such as loss, latency and jitter are considered for future work, along with the construction of detailed profiles for applications taking into account their CPU and memory consumption.

## Figures and Tables

**Table 1:** Different weight configurations of the IPA algorithm.

Name	Bandwidth	Existing Pods	RTT	CPU	Memory
IPA-O	50%	30%	10%	5%	5%
IPA-N	100%	-	-	-	-
IPA-B	70%	30%	-	-	-
IPA-E	50%	50%	-	-	-

## Data Availability

Not applicable.
